# Multiple Roles of Integrin-Linked Kinase in Epidermal Development, Maturation and Pigmentation Revealed by Molecular Profiling

**DOI:** 10.1371/journal.pone.0036704

**Published:** 2012-05-04

**Authors:** David Judah, Alena Rudkouskaya, Ryan Wilson, David E. Carter, Lina Dagnino

**Affiliations:** 1 Department of Physiology and Pharmacology, University of Western Ontario, and Children's Health Research Institute and Lawson Health Research Institute, London, Ontario, Canada; 2 London Regional Genomics Centre, University of Western Ontario, London, Ontario, Canada; 3 Department of Paediatrics, University of Western Ontario, London, Ontario, Canada; University of Munich, Germany

## Abstract

Integrin-linked kinase (ILK) is an important scaffold protein that mediates a variety of cellular responses to integrin stimulation by extracellular matrix proteins. Mice with epidermis-restricted inactivation of the *Ilk* gene exhibit pleiotropic phenotypic defects, including impaired hair follicle morphogenesis, reduced epidermal adhesion to the basement membrane, compromised epidermal integrity, as well as wasting and failure to thrive leading to perinatal death. To better understand the underlying molecular mechanisms that cause such a broad range of alterations, we investigated the impact of *Ilk* gene inactivation on the epidermis transcriptome. Microarray analysis showed over 700 differentially regulated mRNAs encoding proteins involved in multiple aspects of epidermal function, including keratinocyte differentiation and barrier formation, inflammation, regeneration after injury, and fundamental epidermal developmental pathways. These studies also revealed potential effects on genes not previously implicated in ILK functions, including those important for melanocyte and melanoblast development and function, regulation of cytoskeletal dynamics, and homeobox genes. This study shows that ILK is a critical regulator of multiple aspects of epidermal function and homeostasis, and reveals the previously unreported involvement of ILK not only in epidermal differentiation and barrier formation, but also in melanocyte genesis and function.

## Introduction

The skin is the largest organ of the body, and its upper layer, the epidermis, is a barrier that fulfills critical protective and homeostatic functions. The epidermis is mainly composed of several layers of keratinocytes at various stages of differentiation. Specifically, the lowermost basal layer contains keratinocyte stem cells and their undifferentiated committed progeny, whereas the suprabasal layers comprise post-mitotic keratinocytes. Basal keratinocytes adhere to the underlying basement membrane mainly *via* integrins and various hemidesmosomal proteins. Basal keratinocytes are the source of cells needed for epidermal maintenance, renewal and regeneration after injury. These functions complement those of the suprabasal keratinocytes, in which cell-cell adhesion, principally mediated by desmosomes, adherens and tight junctions, imparts to the epidermis its barrier properties (reviewed in [1]).

Keratinocytes in intact epidermis express several integrins, including α6ß4, α3ß1 and α2ß1 [2]. Proper regulation of integrin function is necessary for normal keratinocyte adhesion, proliferation and differentiation. Targeted inactivation of mouse genes encoding α6 or ß4 integrin subunits results in severe epidermal blistering, absence of hemidesmosomes and perinatal lethality [3], [4]. Although skin blistering is less severe in *Intb1*-null mice, these animals can also die perinatally and exhibit abnormalities in keratinocyte proliferation and in hair follicle morphogenesis [5], [6], emphasizing the importance of proper integrin function for epidermal integrity. Integrins bind extracellular matrix (ECM) proteins to contribute to cell adhesion, and to transduce signals that trigger a variety of cellular responses [2]. However, as integrins have no intrinsic catalytic activities, they participate in signaling events through their interactions with a vast array of kinases, as well as scaffold proteins such as ILK.

ILK is a component of focal adhesions [7], was originally identified as a ß1 integrin-associated protein [8], and fulfills various critical roles in epidermal keratinocytes. For example, *Ilk* gene inactivation in the embryonic ectoderm or in the developing epidermis impairs hair follicle morphogenesis and disrupts epidermal attachment to the basement membrane [9], [10]. Further, expression of ILK in keratinocyte stem cells of the hair follicle bulge is essential for normal epidermal regeneration after injury [11]. In cultured keratinocytes, ILK contributes to the development of front-rear polarity and cell-cell junctions, as well as membrane targeting of caveolae [9], [10], [12], [13], [14].

Although substantial efforts have yielded important information regarding the key roles that ILK plays in the skin, the molecular pathways modulated by ILK in this tissue remain poorly defined. A better understanding of these pathways is essential to establish the basis for the severe and multiple, distinct alterations in cutaneous functions consequent to *Ilk* gene inactivation. To address this important issue, we have defined the transcriptome of ILK-deficient epidermis through genome-wide molecular profiling. This approach allowed us to discover novel and unexpected roles of ILK in multiple aspects of epidermal function, development and homeostasis.

## Results and Discussion

### Transcriptional profiling of ILK-deficient epidermis

**Figure 1:**
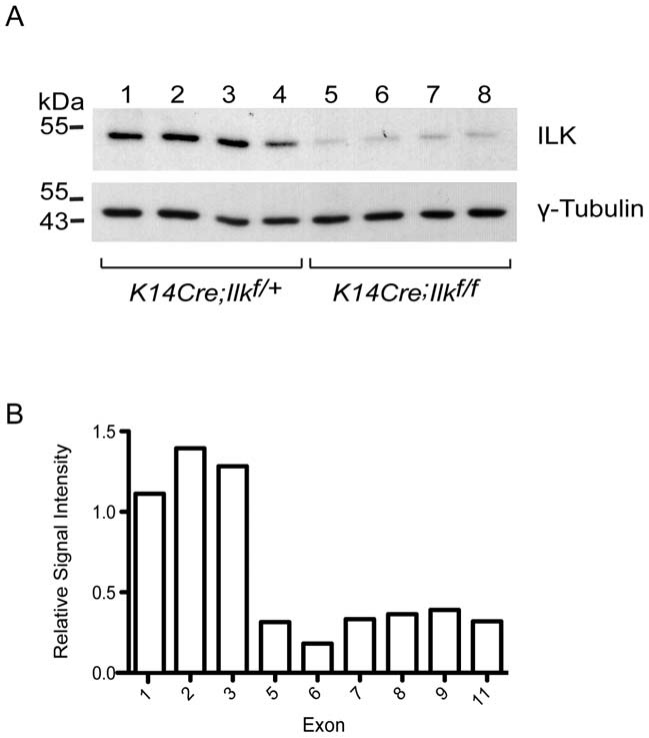
Targeted inactivation of *Ilk* and loss of ILK transcripts and protein in mouse epidermis. (**A**) The skin of 3 day-old *K14Cre-Ilk^f/+^* and *K14Cre-Ilk^f/f^* mice (4 each, from two different litters) was isolated and treated with dispase to separate the epidermis from the dermis. Epidermal lysates were prepared and analyzed by immunoblot with antibodies against ILK or γ-tubulin, used as loading control. (**B**) Total RNA from *K14Cre-Ilk^f/+^* and *K14Cre-Ilk^f/f^* mice (5 each) was used to interrogate GeneChip Mouse Gene 1.0 ST Arrays, and the signal intensity corresponding to individual *Ilk* exons was analyzed. The average intensity values obtained for each exon in RNA from *K14Cre-Ilk^f/f^* epidermis are shown relative to those in *K14Cre-Ilk^f/+^* tissues, which have been set to 1.0.

To define the molecular role of ILK in epidermal function, we analyzed gene expression profiles of ILK-deficient and ILK-expressing epidermis. To this end, we generated mice with epidermis-restricted inactivation of the *Ilk* gene, by breeding *Ilk^f/f^* animals with transgenic mice expressing *Cre* recombinase under the control of the keratin 14 promoter [9]. We prepared protein lysates from ILK-deficient (*K14Cre; Ilk^f/f^*) and ILK-expressing (*K14Cre; Ilk^f/+^*) epidermis from 3 day-old mice, because at this age most animals are still viable, in spite of their severe epidermal alterations [9]. These experiments showed that *K14Cre; Ilk^f/f^* epidermis expresses ILK protein levels that are ≤12% of those found in epidermal tissues from *K14Cre; Ilk^f/+^* mice (Fig. 1A), demonstrating the high efficiency of *Ilk* gene inactivation in our model. We also used epidermal RNA from these animals to interrogate microarrays containing over 750, 000 probe sets that represent 28, 853 mouse genes. Cre-mediated excision of *Ilk^f/f^* alleles is predicted to be associated with presence of transcripts lacking exons 5 through 12, as the floxed *Ilk* alleles contain loxP sites downstream from exons 4 and 12 [15]. Thus, we first investigated the relative signal intensity corresponding to individual exons of the *Ilk* gene from the microarray data. We found high signal values corresponding to exons 1 through 3 in ILK-deficient epidermis, which were similar to those in ILK-expressing tissues. In contrast, the signal intensities of exons 5 through 11 were substantially lower in RNA from ILK-deficient epidermis, further demonstrating the high efficiency of Cre-mediated *Ilk* gene excision (Fig. 1B).

Analysis of the microarray data also revealed statistically significant changes in the expression of a large number of genes in ILK-deficient epidermis. Specifically, 224 genes showed changes in expression that were greater than 2-fold (*P*<0.05, Table S1), and 780 showed 1.5-fold changes (*P*<0.05). Hierarchical clustering of the differentially expressed genes was conducted, which separates the ten samples analyzed into their corresponding genotype groups (Figure S1).

To compare the characteristics of these genes, we grouped them in clusters based on their gene ontology term annotations. In this manner, we established that the largest fractions of differentially expressed genes were present in various categories, including factors involved in signaling, metabolic and developmental processes, in cytoskeletal components and cell adhesion. Significantly, the largest fraction of differentially regulated genes was related to processes central to epidermal structure and/or function, including those involved in hair follicle formation, keratinocyte differentiation and epidermal barrier acquisition, pigmentation, epidermal development and homeostasis.

### Reduced expression of hair follicle genes in ILK-deficient epidermis

**Figure 2:**
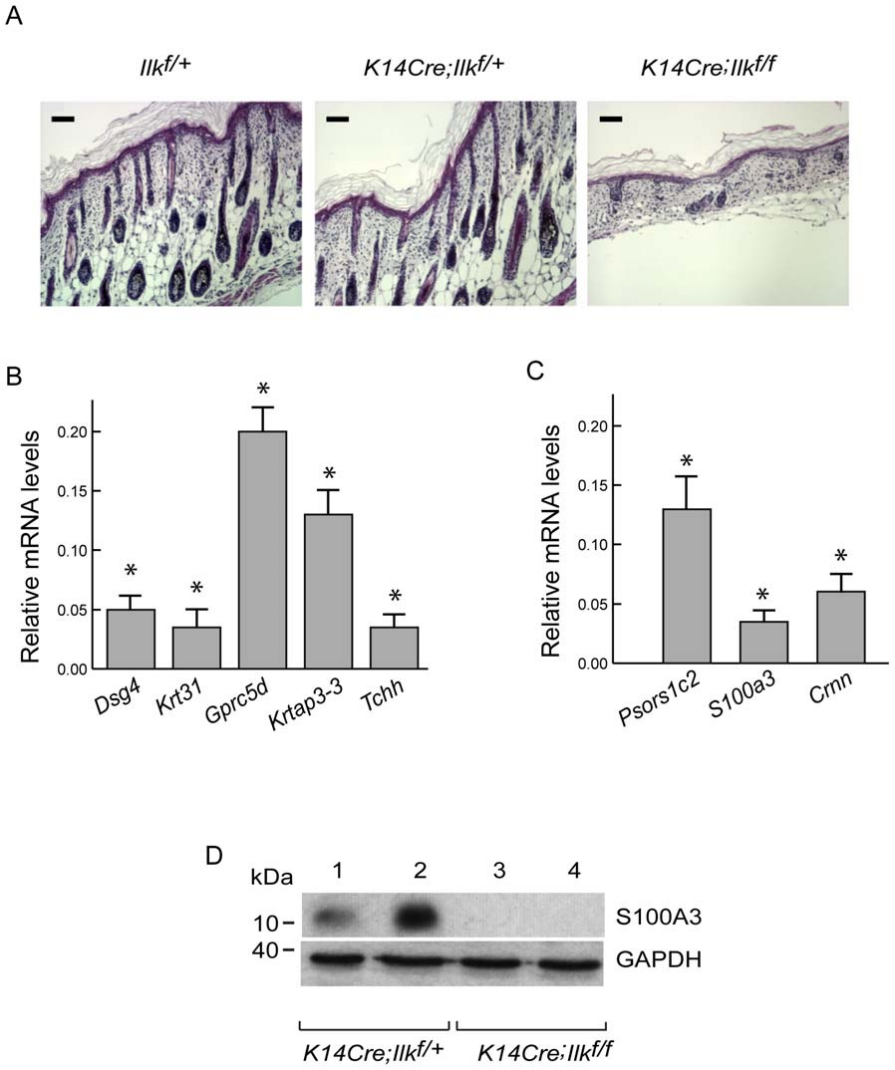
Analysis of hair follicle- and keratinocyte differentiation-associated transcripts in ILK-deficient epidermis. (**A**) Paraffin sections of skin isolated from 3 day-old mice of the indicated genotype were processed for histological analysis, and stained with hematoxylin and eosin. Bar, 40 µm (**B, C**) Expression levels of selected transcripts encoding proteins present in the hair follicle (panel B) or in differentiated keratinocytes (panel C) in ILK-deficient *K14Cre-Ilk^f/f^* epidermis, determined by qPCR. The results are expressed as the mean+SEM (n = 5), and asterisks indicate *P*<0.001 (Student's t test) relative to the corresponding transcript levels in ILK-expressing *K14Cre-Ilk^f/+^* epidermis (set to 1). (**D**) Protein lysates prepared from the skin of 3 day-old mice of the indicated genotypes were resolved by denaturing gel electrophoresis, followed by immunoblot analysis using antibodies against anti-S100A3. The blots were also probed with antibodies against GAPDH, used as loading control.

Hair follicle morphogenesis begins around 14.5 days of gestation (E14.5) in mice. It continues until about 6–7 days after birth (P6–P7), culminating with the formation of fully-grown hair follicles and hair shafts [16], [17]. Epidermis-restricted inactivation of the *Ilk* gene results in impaired hair follicle formation [9], [10]. Histological analyses revealed that the majority of hair follicles in P3 ILK-expressing epidermis have reached stages 5–7, and exhibit well defined hair shafts. In contrast, hair follicles in ILK-deficient epidermis are less abundant and appear severely underdeveloped, with the vast majority remaining at stages 2–4 (Fig. 2A). In addition, skin with ILK-deficient epidermis is thinner at this stage, and shows substantially reduced subcutaneous adipose tissue. Consistent with the follicular abnormalities, microarray analysis revealed that 27% of those transcripts found to be substantially downregulated in ILK-deficient epidermis encode important structural hair follicle elements, such as hair-specific keratins and keratin-associated proteins (e.g. Table S2). Other underrepresented mRNAs important for hair follicle structure and formation included *Dlx3*, which encodes a homeobox transcription factor essential for inner root sheath formation [18], *Dsg4* and *Tchh*, which encode, respectively, desmoglein 4 and trichohyalin.

To confirm the alterations observed from the microarray experiments, we conducted real-time, quantitative polymerase chain reaction (qPCR) analyses on selected transcripts, using RNA preparations from ILK-expressing and ILK-deficient epidermis isolated from additional 3 day-old mice (Fig. 2B and Table 1). Transcript levels of hair keratin *Krt31* and the keratin-associated protein *Krtap3-3* were, respectively, 28- and 8-fold lower in ILK-deficient samples than in normal tissues. Similarly, the orphan G-protein coupled receptor *Gpcr5d*, an important constituent of the differentiated regions of the hair cortex likely involved in the expression of hair keratins [19], was expressed at 5-fold lower levels in ILK-deficient epidermis. Transcripts encoding desmoglein-4, which is an important structural constituent of desmosomes in the hair cuticle and cortex, as well as in the more differentiated spinous layers of the interfollicular epidermis [20], were expressed at ∼18-fold lower levels in ILK-deficient epidermis. Finally, transcripts encoding the inner root sheath protein trichohyalin, which is also found in the terminally differentiated cells of the cornified envelope, were about 28-fold less abundant in the mutant tissues. The reduced abundance of these genes is consistent with the notion that ILK expression in the epidermis is essential for hair follicle maturation past stages 4–5, and confirms the accuracy of our microarray profiling results.

**Table 1:** Validation of the differential expression of selected genes in ILK-deficient epidermis.

			ILK^deficient^/ILK^expressing^ (fold change)	
Class	Gene	Gene Name	Microarray	qPCR
**Hair follicle**				
	*Dsg4*	Desmoglein 4	−7.9	−18.2
	*Krt31*	Keratin 31	−16.1	−28.6
	*Krtap3-3*	Keratin-associated protein 3-3	−18.4	−7.8
	*Gpcr5d*	G protein-coupled receptor family C, Group 5, member D	−14.7	−4.9
	*Tchhc*	Trichohyalin	−16.9	−28.0
**Keratinocyte Differentiation; Psoriasis**				
	*Crnn*	Cornulin	−8.6	−15.5
	*S100A3*	S11 calcium-binding protein A3	−7.5	−21.9
	*Psors1c2*	Psoriasin (S100 A7)	−4.5	−7.6
**Growth factor pathways**				
	*Tgfb2*	Transforming growth factor-ß2	2.9	3.1
	*Ltbp1*	Latent TGF-ß binding protein 1	2.3	1.9
	*Igf1r*	Insulin-like growth factor receptor 1	2.0	2.0
	*Ctgf*	Connective tissue growth factor	2.7	3.7
**Wnt pathway**				
	*Sfrp2*	Secreted frizzle-related protein 2	3.5	2.6
	*Lgr5*	Leucin-rich repeat containing GPCR5	3.1	8.0
**Hedgehog Pathway**				
	*Gli1*	GLI family zinc finger 1	1.9	4.5
	*Gli2*	GLI family zinc finger 2	1.8	5.1
	*Ptch2*	Patched homolog 2	2.3	5.2
	*Bmp7*	Bone morphogenetic protein 7	1.5	3.1
**Miscellaneous**				
	*Hr*	Hairless	2.2	2.6
	*Tyr*	Tyrosinase	2.9	2.0
	*Rhoc*	Ras homolog gene family member C	2.5	3.9

### Role of ILK in expression of genes involved in keratinocyte differentiation and barrier formation

Cells in the lowermost, basal layer of the epidermis exhibit proliferative capacity, and differentiate into suprabasal keratinocytes, characterized by their strong intercellular junctions and post-mitotic character. The suprabasal layers provide the epidermis its barrier properties, *via* assembly of desmosomes and other cell-cell junctions, as well as production of an elaborate network of cross-linked proteins and synthesis of specialized lipids [1]. Significantly, ILK-deficient epidermis exhibited a substantial reduction in the levels of various mRNAs that encode key enzymes and factors necessary for protein cross-linking and lipid biosynthesis. For example, transcript abundance of transglutaminase 3 (*Tgase3*), a major cross-linking enzyme expressed in the spinous and granular layers [21], and of *Prr9*, a member of the small proline-rich proteins that serve as substrates for TGAse [22], were detected at 7- and 20-fold lower levels, respectively, in ILK-deficient epidermis (Table S2). Smaller but significant decreases in the levels of *PPAR-γ*, *Scd2* and *Dhcr24* mRNAs were also detected in ILK-deficient keratinocytes. The latter two genes encode, respectively, stearoyl-coenzyme A desaturase 2, which is involved in the biosynthesis of monounsaturated fatty acids essential for skin permeability barrier [23], and 24-dehydrocholesterol reductase, implicated in synthesis of intercellular lipids of the stratum corneum. Similarly, *PPAR-γ* modulates the transcription of genes involved in epidermal barrier formation, lipid synthesis and sebocyte differentiation, such as cholesterol sulfotransferase [24].

In addition to desmoglein 4, other components of the cornified envelope were substantially downregulated in the absence of ILK. Cornulin is a marker of late epidermal differentiation found in the granular and in the cornified layers [25], and its 10-fold lower abundance in ILK-deficient epidermis was confirmed by qPCR (Fig. 2C, Table 1). Transcripts encoding the Ca^2+^-binding protein S100A3, involved in maturation of the epidermis and appendages such as hair follicles [26], were 15-fold less abundant as measured by qPCR, and no detectable S100A3 protein was found in lysates from *K14Cre; Ilk^f/f^* skin (Fig. 2C and 2D, Table 1). *Ilk* gene inactivation is also associated with altered distribution of the suprabasal marker loricrin [10], [27]. Thus, ILK plays a broad and important positive modulatory role in keratinocyte differentiation and acquisition of epidermal barrier function.

### Nexus between ILK expression and psoriasis

Psoriasis is a chronic cutaneous inflammatory disease with genetic and auto-immune components. Psoriatic skin is scaly and thickened, due to marked increases in keratinocyte proliferation, accompanied by altered differentiation [28]. Analyses of the psoriatic transcriptome have identified a large number of genes upregulated in these lesions. Significantly, a subset of these genes is downregulated in ILK-deficient epidermis (Fig. 2C, Table 1 and Table S2). Examples of these genes include *SerpinB3A, S100A3, S100A6, Sprr20*, and *Akr1c18*. Of particular interest is the downregulation of *Psors1c2*, which encodes psoriasin (also termed S100A7), which is important in promoting anti-microbial activity, survival and responses to inflammatory stimuli in keratinocytes. Psoriasin is also upregulated in a variety of epithelial tumours, including oral carcinomas, in which it increases the invasive capabilities of transformed cells [29]. These findings suggest a potential link between ILK expression and disorders that involve aberrant keratinocyte proliferation, and will be an important area for future research.

### Modulation of genes implicated in responses to injury and growth factor signaling

**Figure 3:**
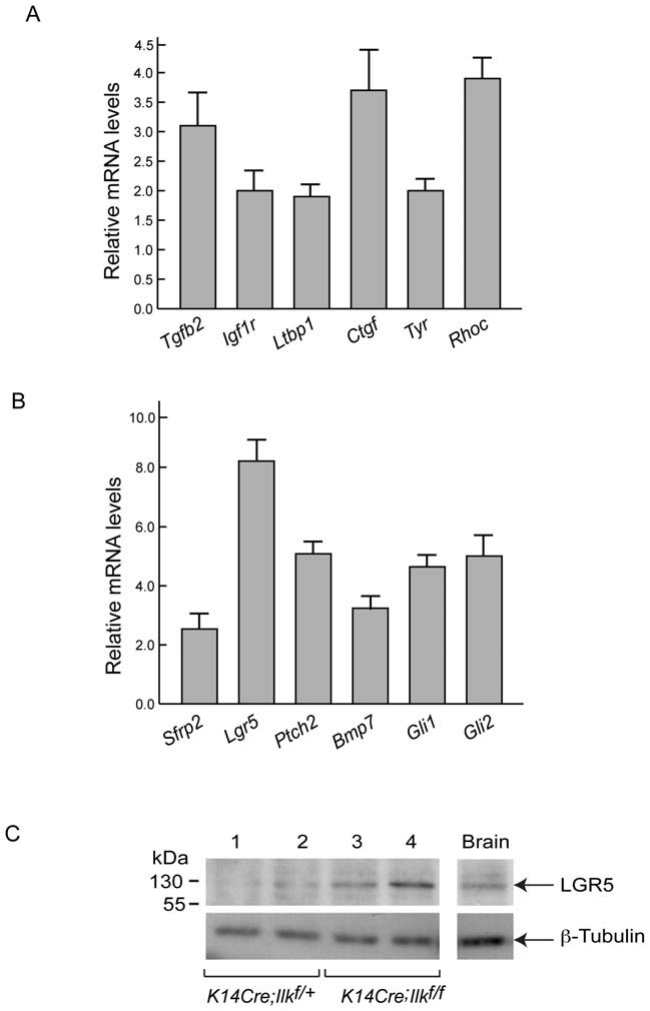
Altered expression of genes encoding growth factors and developmental regulators in ILK-deficient epidermis. (**A, B**) Expression levels of selected transcripts encoding proteins associated with growth factors or pigmentation (panel A), or key factors in developmental epidermal pathways (panel B) in ILK-deficient *K14Cre-Ilk^f/f^* epidermis, determined by qPCR. The results are expressed as the mean+SEM (n = 5). The differences in expression in ILK-deficient epidermis for each of transcripts shown reached statistical significance (*P*<0.001, Student's t test) relative to the corresponding transcript levels in ILK-expressing *K14Cre-Ilk^f/+^* epidermis (set to 1). (**C**) Protein lysates prepared from the skin of 3 day-old *K14Cre-Ilk^f/f^* or *K14Cre-Ilk^f/+^* mice (two animals of each genotype) were resolved by denaturing gel electrophoresis, followed by immunoblot analysis using antibodies against LGR5, or γ-tubulin, used as loading control. Lysates prepared from wild type mouse brain were used as a positive control for LGR5 expression.

ILK-deficient epidermis exhibits regions with loss of integrity in the dermal-epidermal junction and microblister formation, particularly in friction-prone areas [9], [10]. As early as 2–3 days after birth, ILK-deficient epidermis develops erosions, acanthosis, extensive edema and inflammation. Consistent with these alterations, our profiling studies demonstrate up to 6-fold upregulation of various genes that play key roles in maintenance of epidermal homeostasis and repair following injury, particularly those implicated in various growth factor pathways (Figure 3A, Table 1 and Table S2). Transcripts that encode transforming growth factor (TGF)-ß 2 and 3 isoforms, which are expressed by keratinocytes and play important modulatory roles for dermal fibroblasts [30], are upregulated. We also found increased levels of *Ltbp1* mRNA, which is consistent with previous finding demonstrating that expression of TGF-ß is coordinately regulated with that of latent TGF-β-binding proteins [31]. The increases in TGF-ß expression appear to have functional implications, as we also detected elevated levels of direct TGF-ß targets, such as *Igfbp4* and *Igfpb7*, which encode the insulin-like growth factor (IGF)-binding proteins 4 and 7. IGF plays important roles in keratinocyte proliferation, survival, hair follicle growth and epidermal repair following mechanical or UV-induced damage [32], [33]. ILK-deficient epidermis also exhibits increased levels of IGF receptor 1 (*Igfr1*) and FGF receptor 1 (*Fgfr1*) mRNAs. FGFR1 functions coordinately with IGF receptor activation, playing key roles in cutaneous homeostasis, inflammation and repair pathways [34]. Epidermal ILK-deficiency also resulted in elevated levels of transcripts encoding Connective Tissue Growth Factor (CTGF), a member of the CCN family of matricellular proteins expressed by keratinocytes and fibroblasts. CTGF is upregulated by TGF-ß and other inflammatory cytokines, and is central for regenerative and fibrotic processes [30]. Whether ILK directly regulates all of these genes remains to be determined, but it is clear that its absence results in cutaneous alterations with broad, pleiotropic effects on multiple pathways that are themselves key for normal function, repair and/or maintenance of the skin.

### Epidermal ILK deficiency is associated with increased expression of genes involved in Wnt signaling

The Wnt signaling pathway plays important roles in patterning and development of a wide variety of tissues, in stem cell maintenance and in carcinogenesis [35]. Wnt signals are required for embryonic hair follicle development and postnatal anagen growth [36]. A link between Wnt and ILK has been proposed in some cell types, based on the observation that exogenous ILK expression in intestinal or mammary epithelial cells reportedly induced activation of the downstream Wnt effectors ß-catenin and Lymphoid Enhancer Factor (LEF)-1 [37]. Similarly, ILK is required for activation of Wnt pathways during somite formation in chick embryos [38]. In contrast, *Ilk* gene expression does not appear to be required for Wnt signaling in the post-natal hair follicle, as its inactivation in stem cells of the hair follicle bulge does not cause detectable alterations in anagen hair growth [11]. The results of our microarray further confirm the notion that ILK is not required for Wnt signaling in at least some aspects of epidermal appendage growth. In fact, ILK would appear to have a moderate negative modulatory effect on Wnt-activated pathways, as we observed upregulation of several direct Wnt targets in ILK-deficient P3 epidermis, including *Sfrp2* (3.5-fold), *Lgr5* (3.1-fold), *Lgr6* (2-fold), *Wif1* (1.6-fold), and *Dkk3* (2-fold, Fig. 3B, Table 1, Figure S2 and Table S2). Secreted Wnt modulators, such as SFRP2, provide a mechanism for fine tuning canonical Wnt responses and planar cell polarity processes, by binding to Wnt ligands and locally limiting their actions [35].

The G protein-coupled receptor LGR5 is both a target and a modulator of Wnt responses. LGR5 binds to R-spondin, and cooperates with Wnt to activate ß-catenin/LEF-1 and planar cell polarity (PCP) signaling [39], [40]. In the epidermis, LGR5 is found in the E18.5 developing outer root sheath, in newborn hair follicles and in the hair follicle bulge stem cells of adult mice [41]. In the latter, LGR5 controls stem cell activity, is important for hair follicle regeneration during anagen, and constitutes a key marker for hair follicle stem cells. We confirmed the increases in *Lgr5* mRNA by qPCR (Table 1), and found that they are accompanied by increases at the protein level (Fig. 3B and 3C). At present, the functional significance of these changes in LGR5 on hair follicle biology is not clear, as *K14Cre;Ilk^f/f^* mice succumb before hair follicle development is completed. This will be an important area for future research.

### Epidermal ILK deficiency is associated with increased expression of sonic hedgehog (Shh) targets

During morphogenesis of the epidermis and its appendages, Shh produced by the dermal mesenchyme stimulates keratinocytes, participating in hair follicle formation. Following birth, Shh is important for anagen hair follicle growth (reviewed in [42]). The Shh pathway is also positively modulated by Wnt ligands in developing hair follicles. Shh binding to its receptors, PTCH 1 and 2, and ultimately results in activation of GLI transcription factors, creating a positive feedback loop in which GLI proteins further activate transcription of *Ptch* and *Gli* genes [42]. Associated with the abnormal development of ILK-deficient hair follicles, our analysis also found evidence of increased activation of Shh signaling in P3 tissues. In particular, we found increased mRNA levels of the direct Shh downstream targets *Ptch2, Gli1, Gli2, Mycn, Bcl2*, and *Bmp7* [43] (Fig. 3B, Table 1, Figure S2 and Table S2). Shh signaling is important for invagination of stage-2 hair follicles [44], and the evidence of Shh signaling in ILK-deficient epidermis is consistent with the observed ability of ILK-deficient hair follicles to develop at least to stages 2–4. The increased levels of Shh-modulated targets in ILK-deficient skin from 3 day-old mice may contribute to the abnormal hair follicle development observed. Alternatively, it may reflect the arrest in hair follicle growth at stages 2–4 observed in the postnatal ILK-deficient epidermis, as during these early stages the Wnt and Shh pathways are active, but their activation decreases during later stages of hair follicle morphogenesis, which have been reached at P3 in ILK-expressing epidermis.

### Melanocyte signature genes in ILK-deficient epidermis

In the mouse embryo, neural crest cells migrate towards the ectoderm from E8.5 to E12, and colonize the epidermis and developing hair follicles as melanoblasts. Around the time of birth, melanoblasts differentiate into melanocytes, which remain in the interfollicular epidermis or home in the hair bulbs [45]. Although melanogenic gene expression begins with the activation of *Sox10* in the neural crest cells at E8.5–E9.0, fully differentiated melanocytes that produce pigment granules only arise around birth. *Sox10* expression is maintained in melanoblasts and melanocyte stem cells that home in the hair follicle bulge in post-natal skin.

SOX10 directly activates another key melanogenic gene, *Mitf*, around E9.5–E10.0. These two transcription factors cooperate to induce expression of key melanin-producing enzymes, such as tyrosinase, around E16.5. Later on, the perinatal period is characterized by activation of melanin biosynthesis and uptake of melanin-containing melanosomes by keratinocytes [46], [47]. In mice, progressive loss of interfollicular epidermal melanocytes occurs during the first few weeks after birth [48].

Although no major differences in appearance are evident at birth, by P3 *K14Cre;Ilk^f/+^* mice show substantial cutaneous pigmentation, whereas *K14Cre;Ilk^f/f^* mice do not (Figure S3). In apparent contradiction with these observations, our microarray and qPCR analyses indicate increases in the abundance of mRNAs encoding SOX10, MITF, TYR and TYRP1 in ILK-deficient epidermis (Table 1, Figure S4 and Table S2). The presence of these transcripts suggests that lack of ILK in keratinocytes does not interfere with the early homing of melanoblasts to the skin or with expression in these cells and/or their descendants of rate-limiting enzymes for melanin production, such as TYR and TYRP1. Full differentiation and proliferation of melanocytes requires multiple factors, including some that are secreted by keratinocytes [48]. Thus, important areas for future research will include determining whether the apparent hypopigmentation of *K14Cre;Ilk^f/f^* skin results from altered keratinocyte-melanocyte interactions that lead to reduced melanin biosynthesis and/or impaired melanosome uptake by keratinocytes.

In summary, genome-wide analyses demonstrates that ILK is essential for proper development of epidermal appendages, for barrier formation and keratinocyte maturation, as well as pigmentation and regenerative processes.

## Materials and Methods

### Antibodies and reagents

Antibodies and their sources are as follows: ß-tubulin (E7, Developmental Studies Hybridoma Bank, University of Iowa), ILK (mouse monoclonal, 611802, Transduction Laboratories, Lexington, KY, or rabbit polyclonal, 1979-1, Epitomics, Burlingame, CA), S100A3 (rabbit polyclonal, 12343-1-AP, ProteinTech Group, Chicago, IL), LGR5 (rabbit polyclonal, AP2745f, Abgent, San Diego, CA), Glyceraldehyde-3-phosphate dehydrogenase (GAPDH; mouse monoclonal, CSA-335, Assay Designs, Ann Arbor, MI). Dispase II (04 942 078 001) was purchased from Roche Diagnostics (Indianapolis, IN).

### Mouse strains

The mouse strains used were: *Ilk^tm1Star^*, with engineered *loxP* sites downstream from exons 4 and 12 of the *Ilk* gene (hereafter termed *Ilk^f/f^*; [15]), and *Tg(KRT14-cre)1Amc/J* (hereafter termed *K14Cre*; Stock No. 004782, The Jackson Laboratory, Bar Harbor, Maine) [49]. These two strains were bred to produce animals hemizygous for the *KRT14-cre* allele, and either homozygous or heterozygous for the floxed *Ilk* allele (hereafter termed, respectively, *K14Cre; Ilk^f/f^* or *K14Cre; Ilk^f/+^*). Mice were genotyped as previously described [9].

### Ethics statement

All protocols for mouse experimentation were approved by the University of Western Ontario Animal Use Care Committee (Approved Protocol No. 2007-005, 2011), in accordance with regulations and guidelines from the Canadian Council on Animal Care.

### RNA preparation and processing

Skins from 3 day-old mice were harvested and digested with dispase diluted in phosphate-buffered saline (PBS; 8 mg/ml, final) for 1 h at 37°C. After digestion, the epidermis was mechanically separated from the dermis. RNA from 25-mg fragments was extracted and purified using RNeasy minikits (Qiagen, Louisville, KY), following the manufacturer's instructions. RNA quantity and quality for microarray experiments and qPCR were assessed using an Agilent 2100 Bioanalyzer (Agilent Technologies, Palo Alto, CA) and RNA 6000 Nano kit (Caliper Life Sciences, Hopkinton, MA). Only samples with RNA integrity numbers ≥8 were used. For probe preparation, cDNA synthesis and *in vitro* transcription from 5 µg total RNA were done with an Ambion WT Expression Kit for Affimetrix GeneChip Whole Transcript WT Expression Arrays (Ambion, Foster City, CA), as per manufacturer's protocols.

### Microarray hybridization and bioinformatics analyses

Probes corresponding to RNA samples isolated from five diferent *K14Cre; Ilk^f/f^* and five *K14Cre; Ilk^f/+^* mice were used to hybridize a total of ten GeneChip Mouse Gene 1.0 ST Arrays (Affymetrix, Foster City, CA). Gene array hybridization and analyses were conducted at the London Regional Genomics Centre of the University of Western Ontario, according to the Affymetrix GeneChip Expression Technical Manual. Following hybridization, washing and staining, arrays were scanned with a GeneChip Scanner 3000 (Affymetrix).

Gene expression levels were analyzed using Partek Genomics Suite v6.5. The microarray data were first processed using the GC (guanine and cytosine) Robust Multi-chip Averaging (GC-RMA) algorithm from Genespring GX*. The data were then additionally filtered by retaining probe sets with expression values ≥50. Genes with expression values that were indistinguishable from background intensity were filtered out as they constitute genes not expressed in keratinocytes, or expressed at very low levels, such that their measurements were not reliable. Subsequent comparisons were filtered using a 1.5- and 2-fold change thresholds. The data sets were normalized using RMA with RMA Express software v.0.4.1. [50] The data were processed with background adjustment and quantile normalization parameters. Expression values were logarithmically transformed, and used for Gene Set Enrichment Analysis (GSEA), using GSEA v.2.0 software. *P*-values were determined using GC-RMA and ANOVA. Differential gene expression between ILK-expressing and ILK-deficient pools was established based on fold-change and *P*-values. Probe sets with a fold change ≥1.5 or ≤−1.5 and a *P*-value lower than 0.05 were defined to be differentially regulated and selected for further analyses. In compliance with MIAME standards, data files have been deposited into the NCBI Gene Expression Omnibus repository (GEO accession No. GSE35129). Gene Ontology terms were determined using Partek Genomics Suite v 6.5 (Partek Inc., St. Louis, MO), and pathway maps for differentially regulated genes were generated based on Ingenuity Pathway Analysis (Ingenuity Systems, Redwood City, CA).

### Quantitative reverse-transcription PCR (qPCR)

Total epidermal RNA (100 ng/sample) was reverse-transcribed using Superscript II (Invitrogen, Carlsbad, CA) following the manufacturer's instructions. qPCR assays were conducted with oligonucleotide primer sequences shown in Table S3, and cDNA corresponding to 25 ng of initial RNA, using SYBR Green Quantitect PCR kits (Qiagen), as per manufacturer's protocol. Triplicate cDNA samples from epidermal RNA isolated from five *K14Cre; Ilk^f/f^* and five *K14Cre; Ilk^f/+^* mice (different animals from those used for the microarray interrogation experiments) were amplified for 40 cycles (95°C for 15 sec; 50°C for 1 min per cycle) in a 7900HT Fast Real-Time PCR system (Applied Biosystems, Foster City, CA). Initial transcript count (N_o_) values were calculated using raw fluorescence data and LinRegPCR. The results were normalized to the expression of the housekeeping genes *Rpl27* and *Rps29*, which encode ribosomal protein L27 and S29, respectively [51]. Fold changes were calculated comparing ILK-expressing versus ILK-deficient epidermis.

### Histology, protein isolation and immunoblot analysis

Mouse tissues were dissected and fixed in 4% paraformaldehyde and embedded in paraffin. Tissues were sectioned at 8 µm. Hematoxylin-eosin staining was conducted according to standard procedures. Protein lysates were prepared and analyzed by immunoblot, as previously described [13], [52].

## Supporting Information

Figure S1 **Hierarchical clustering of differentially expressed genes in ILK-deficient epidermis.** Genes expressed with at least 1.5 fold change between ILK-expressing and ILK-deficient epidermis are shown. In the clustering heat map, red and blue indicate, respectively, up- and downregulation. In the sample clustering dendogram, green indicates epidermis from *K14Cre;Ilk^f/+^* mice, whereas orange represents *K14Cre;Ilk^f/f^* epidermis.
(TIF)

Figure S2 **Activation of the Wnt and the Hedgehog pathways in ILK-deficient epidermis.** Increased levels of transcripts from target genes of the Wnt and hedgehog pathways. Upregulated transcripts are shown in the pink boxes. The numbers indicate the fold increase in ILK-deficient epidermis for each transcript shown, relative to levels found in ILK-expressing epidermal tissue.
(TIF)

Figure S3 **Phenotypic abnormalities in mice with ILK-deficient epidermis.** Three day-old mice of the indicated genotype are shown. Notice the reduced size and virtual lack of visible pigmentation in the ILK-deficient epidermis of the *K14Cre; Ilk^f/f^* mouse.
(TIF)

Figure S4 **ILK modulation of melanogenesis.** The Wnt pathway is activated in ILK-deficient epidermis. This and other, as yet unidentified, pathways likely result in the observed up-regulation of *Mitf* expression. Increased *Mitf* mRNA levels are also associated with enhanced abundance of transcripts encoding TYR and TYRP-1, two rate-limiting enzymes in the production of melanin. Numbers in pink boxes indicate the fold-increase in ILK-deficient epidermis for each transcript shown.
(TIF)

Table S1 **List of Genes whose expression is >2.0-fold different in ILK-deficient epidermis.**
(XLSX)

Table S2 **Selected genes differentially expressed in ILK-deficient epidermis.**
(DOCX)

Table S3 **Sequences of primers used for qPCR experiments.**
(DOCX)
