# Supplementary figures and images for: Multiple Roles of Integrin-Linked Kinase in Epidermal Development, Maturation and Pigmentation Revealed by Molecular Profiling
Source: PLoS One. 2012 May 4;7(5):e36704. doi: 10.1371/journal.pone.0036704 (PMC3344928; doi:10.1371/journal.pone.0036704)

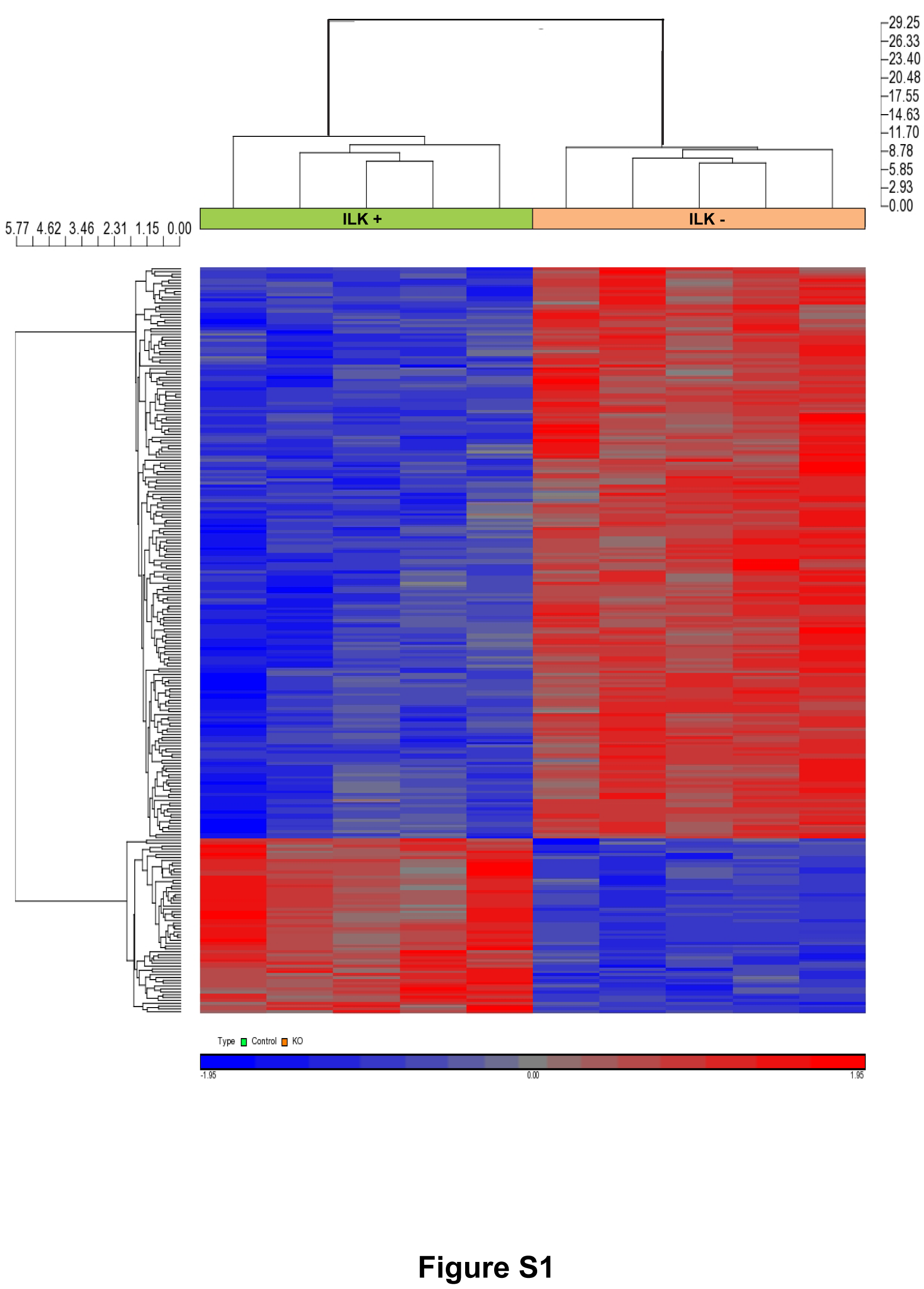

Supplement: Figure S1 — Hierarchical clustering of differentially expressed genes in ILK-deficient epidermis. Genes expressed with at least 1.5 fold change between ILK-expressing and ILK-deficient epidermis are shown. In the clustering heat map, red and blue indicate, respectively, up- and downregulation. In the sample clustering dendogram, green indicates epidermis from K14Cre;Ilkf/+ mice, whereas orange represents K14Cre;Ilkf/f epidermis. (TIF) [file pone.0036704.s001.tif]

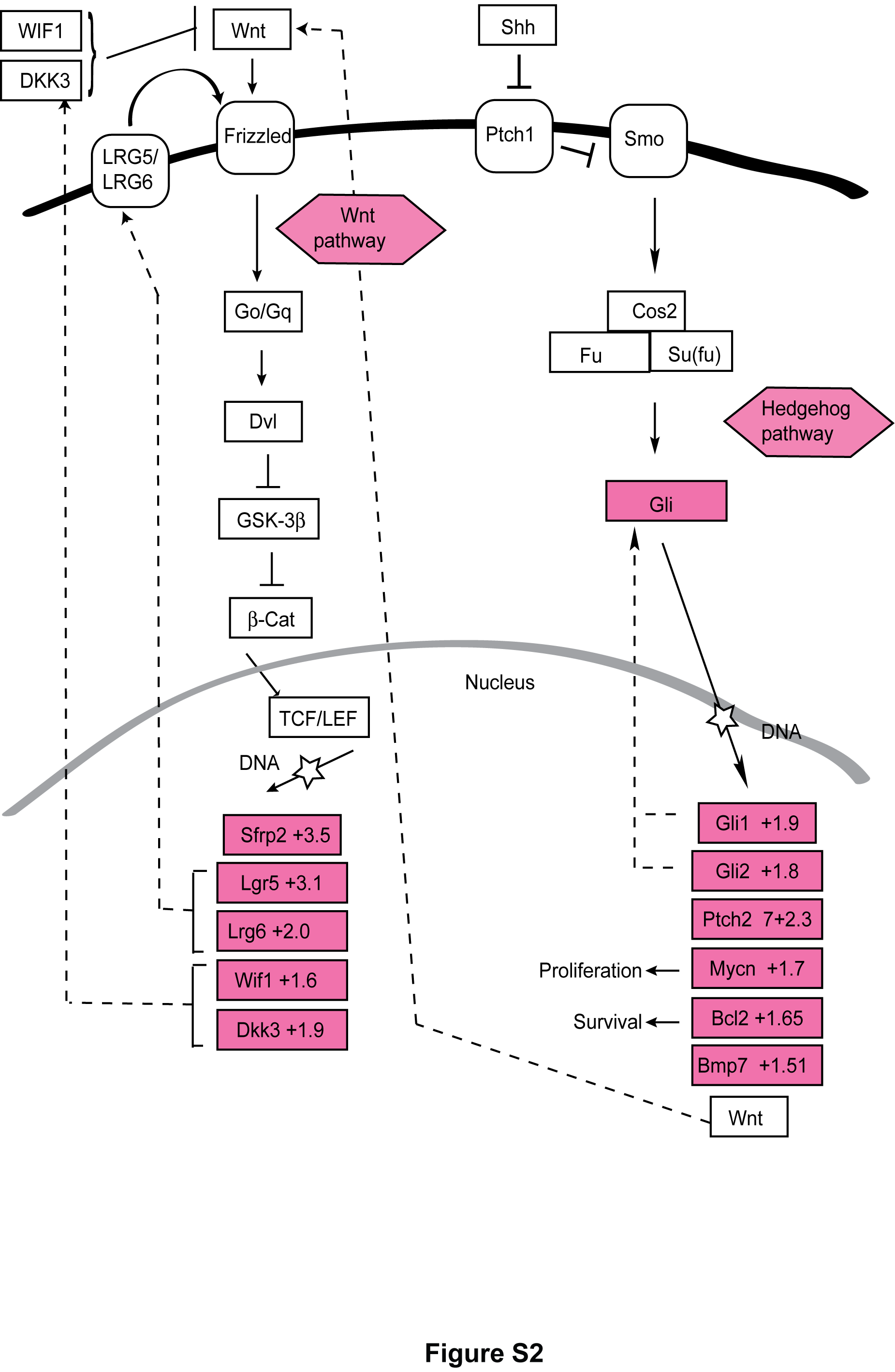

Supplement: Figure S2 — Activation of the Wnt and the Hedgehog pathways in ILK-deficient epidermis. Increased levels of transcripts from target genes of the Wnt and hedgehog pathways. Upregulated transcripts are shown in the pink boxes. The numbers indicate the fold increase in ILK-deficient epidermis for each transcript shown, relative to levels found in ILK-expressing epidermal tissue. (TIF) [file pone.0036704.s002.tif]

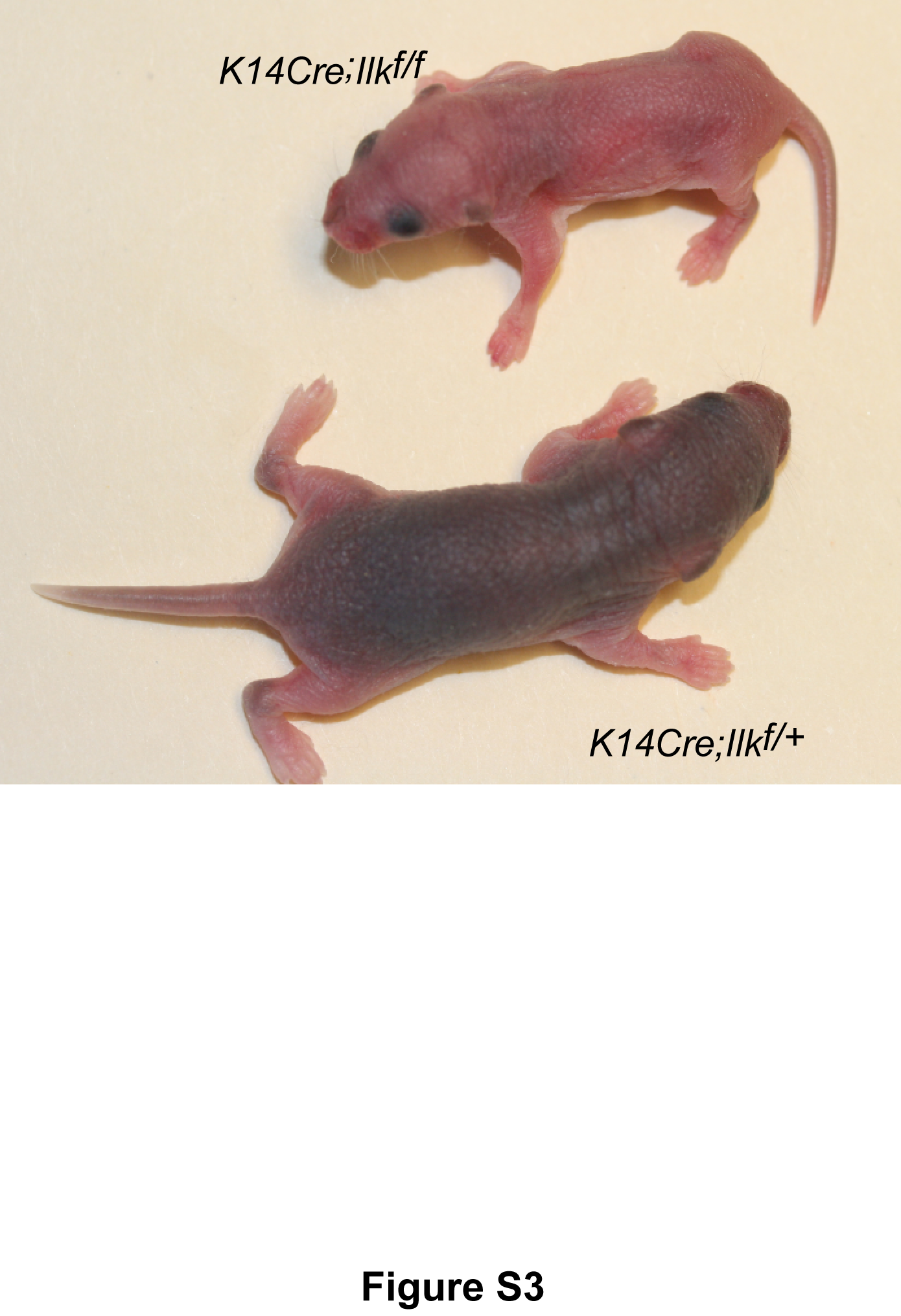

Supplement: Figure S3 — Phenotypic abnormalities in mice with ILK-deficient epidermis. Three day-old mice of the indicated genotype are shown. Notice the reduced size and virtual lack of visible pigmentation in the ILK-deficient epidermis of the K14Cre; Ilkf/f mouse. (TIF) [file pone.0036704.s003.tif]

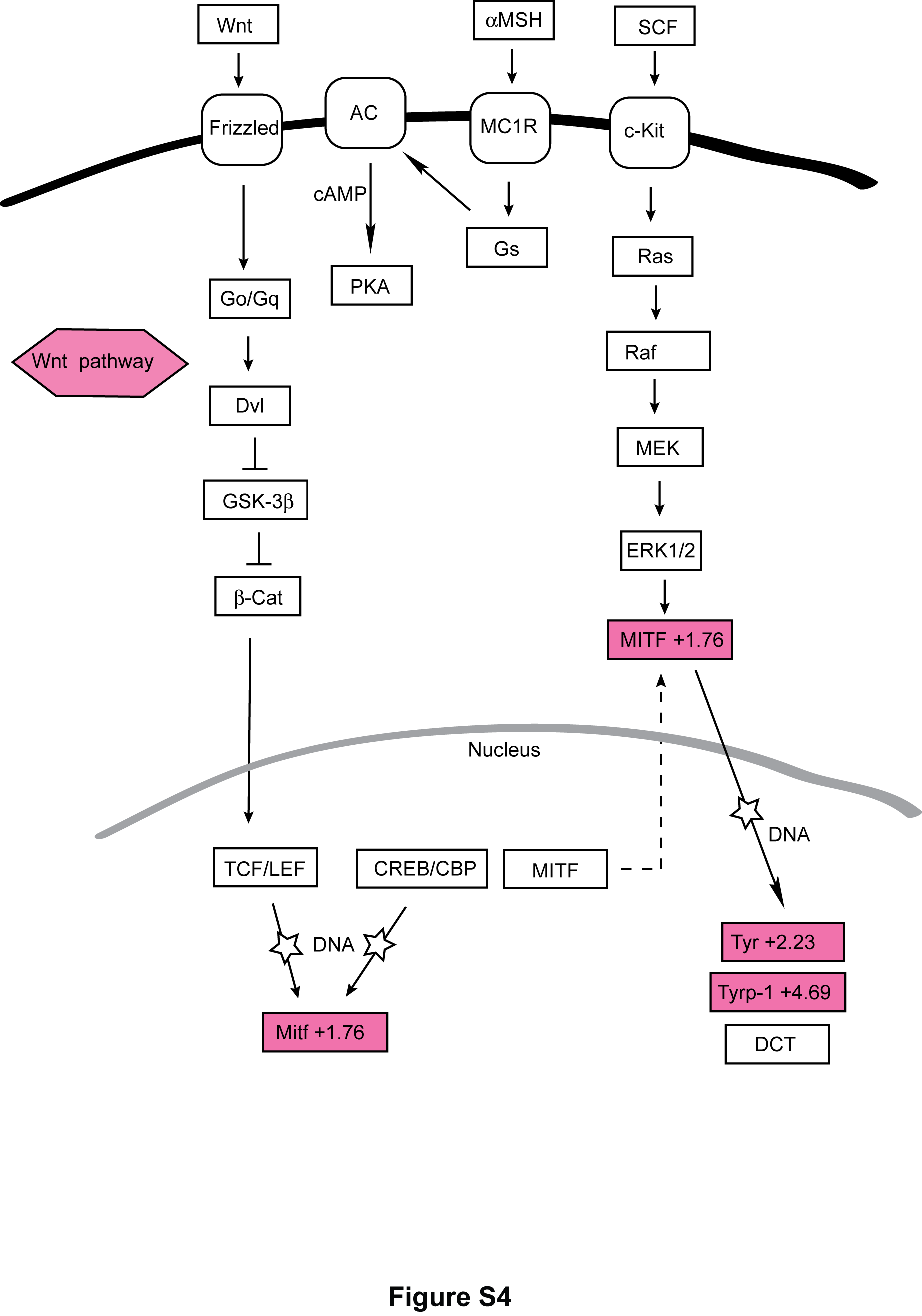

Supplement: Figure S4 — ILK modulation of melanogenesis. The Wnt pathway is activated in ILK-deficient epidermis. This and other, as yet unidentified, pathways likely result in the observed up-regulation of Mitf expression. Increased Mitf mRNA levels are also associated with enhanced abundance of transcripts encoding TYR and TYRP-1, two rate-limiting enzymes in the production of melanin. Numbers in pink boxes indicate the fold-increase in ILK-deficient epidermis for each transcript shown. (TIF) [file pone.0036704.s004.tif]
