# Supplementary material for: Multiple Roles of Integrin-Linked Kinase in Epidermal Development, Maturation and Pigmentation Revealed by Molecular Profiling
Source: PLoS One. 2012 May 4;7(5):e36704. doi: 10.1371/journal.pone.0036704 (PMC3344928; doi:10.1371/journal.pone.0036704)
Supplement: Table S2 — Selected genes differentially expressed in ILK-deficient epidermis. (DOCX) [file pone.0036704.s006.docx]

| **Class** | **Gene** | **Gene Name** | **Accession No.*** | **Fold change**** |
| --- | --- | --- | --- | --- |
| **Hair follicle** | | | | |
|  | *Dsg4* | Desmoglein 4 | NM 181564 | -7.95 |
|  | *Krt31* | Keratin 31 | NM 010659 | -16.09 |
|  | *Krtap3-3* | Keratin-associated protein 3-3 | NM 025524 | -18.14 |
|  | *Gpcr5d* | G protein-coupled Receptor family C,  Group 5, member D | NM 053118 | -14.71 |
|  | *Tchh* | Trichohyalin | ENSMUST00000064257 | -16.88 |
|  | *Krtap4-16* | Keratin-associated protein 4-16 | NM 001013823 | -10.06 |
|  | *Krtap7-1* | Keratin-associated protein 7-1 | NM 027771 | -35.97 |
|  | *Krtap15* | Keratin-associated protein 15 | NM 013713 | -2.43 |
|  | *Dlx3* | Distal-less homeobox 3 | NM 010055 | -1.74 |
|  | *Hoxc5* | Homebox C5 | NM 175730 | -1.69 |
| **Keratinocyte Differentiation, Psoriasis** | | | | |
|  | *Crnn* | Cornulin | NM 001081200 | -8.6 |
|  | *S100A3* | S100 calcium-binding protein 3 | NM 011310 | -7.5 |
|  | *Psors1c2* | Psoriasin (S100 A7) | NM 020576 | -4.5 |
|  | *Prr9* | Proline-rich 9 | NM175424 | -20.25 |
|  | *Pparg* | Peroxisome proliferator activated receptor gamma | NM 001127330 | -1.69 |
|  | *S100A6* | S100 calcium binding protein A6 (calcyclin) | NM 011313 | -1.98 |
|  | *Dhcr24* | 24-Dehydrocholesterol reductase | NM 053272 | -1.63 |
|  | *Scd2* | Stearoyl-coenzyme A desaturase 2 | NM 009128 | -1.60 |
|  | *S100A7a* | S100 calcium-binding protein A7A | NM 199422 | -1.93 |
|  | *Serpinb3a* | Serine peptidase inhibitor, Class B | NM 009126 | -1.57 |
|  | *Akr1c18* | Aldo-ketoreductase family 1, member C18 | NM 134066 | -1.87 |
| **Growth factor pathways** | | | | |
|  | *Tgfb2* | Transforming growth factor -β2 | NM 009367 | 2.98 |
|  | *Tgfb3* | Transforming growth factor -β3 | NM 009368 | 1.62 |
|  | *Ltbp1* | Latent TGF-β binding protein 1 | NM 0 | 2.3 |
|  | *Igf1r* | Insulin-like growth factor receptor 1 | NM 010513 | 2.00 |
|  | *Ctgf* | Connective tissue growth factor | NM 010217 | 2.73 |
|  | *Fgfr1* | Fibroblast growth factor receptor 1 | NM 010206 | 1.7 |
|  | *Pdgfc* | Platelet-derived growth factor, C | NM 19971 | 1.65 |
|  | *Igfbp7* | Insulin-like growth factor binding protein7 | NM 001159518 | 1.81 |
|  | *Igfbp4* | Insulin-like growth factor binding protein4 | NM 010517 | 1.69 |
|  | *Tgfb1i1* | TGF-beta induced transcript 1 | NM 009365 | 1.68 |
| **Wnt pathway** | | | | |
|  | *Sfrp2* | Secreted frizzled-related protein 2 | NM 009144 | 3.5 |
|  | *Lgr5* | Leucine-rich repeat containing GPCR 5 | NM 10195 | 3.1 |
|  | *Wif1* | Wnt inhibitory factor 1 | NM 011915 | 1.65 |
|  | *Lgr6* | Leucine-rich repeat containing GPCR 6 | NM 001033409 | 2.00 |
|  | *Dkk3* | Dickkopf homolog 3 | NM 015814 | 1.98 |
|  |  |  |  |  |
| **Shh pathway** | | | | |
|  | *Gli1* | GLI family zinc finger 1 | NM 010296 | 1.89 |
|  | *Gli2* | GLI family zinc finger 2 | NM 001081125 | 1.84 |
|  | *Ptch2* | Patched homolog 2 | NM 008958 | 2.32 |
|  | *Bmp7* | Bone morphogenetic protein 7 | NM 007557 | 1.51 |
|  | *Mycn* | Myc-N | NM008709 | 1.66 |
|  | *Bcl2* | B-cell leukemia/lymphoma 2 | NM 009741 | 1.65 |
| **Miscellaneous** | | | | |
|  | *Tyrp1* | Tyrosinase-related protein 1 | NM 031202 | 4.69 |
|  | *Tyr* | Tyrosinase | NM 021877 | 2.23 |
|  | *Mitf1* | Microphthalmia-associated transcription factor | NM 001113198 | 1.76 |
|  | *Sox10* | SRY-box containing gene 10 | NM 011437 | 1.61 |
|  | *Rhoc* | Ras homolog gene family member C | NM007484 | 3.9 |

* The accession numbers provided are unique identifiers for the complete RefSeq record submitted to GenBank, and correspond to cDNA sequences constructed from the mRNA encoded by each gene presented in the Table.

****** The data are expressed relative to transcript abundance in ILK-expressing epidermis, which has been set to 1.
