# Supplementary material for: Multiple Roles of Integrin-Linked Kinase in Epidermal Development, Maturation and Pigmentation Revealed by Molecular Profiling
Source: PLoS One. 2012 May 4;7(5):e36704. doi: 10.1371/journal.pone.0036704 (PMC3344928; doi:10.1371/journal.pone.0036704)
Supplement: Table S3 — Sequences of primers used for qPCR experiments. (DOCX) [file pone.0036704.s007.docx]

**TABLE S3**

| Gene Symbol | Genebank ID | Forward Primer | Reverse Primer |
| --- | --- | --- | --- |
| *Dsg4* | NM_181564 | 5’-AACCTGGCAGACTGTCAGAAG-3’ | 5’-GTAAGGTGGCCGATCAATCCC-3’ |
| *Sfrp2* | NM_009144 | 5’-CGTGGGCTCTTCCTCTTCG-3’ | 5’-ATGTTCTGGTACTCGATGCCG-3’ |
| *Gprc5d* | NM_053118 | 5’-TACCATATTGCTACTCCTGGCA-3’ | 5’-AGGCAAAAGTAAGTCCGAAGAG-3’ |
| *Hr* | NM_021877 | 5’-CCCCTGTGAACGGCATTGT-3’ | 5’-CCCCTCCAAAAGGGAGCAG-3’ |
| *Tgfb2* | NM_009367 | 5’-TCGACATGGATCAGTTTATGCG-3’ | 5’-CCCTGGTACTGTTGTAGATGGA-3’ |
| *Ptch2* | NM_008958 | 5’-CTCCGCACCTCATATCCTAGC-3’ | 5’-TCCCAGGAAGAGCACTTTGC-3’ |
| *S100A3* | NM_011310 | 5’-CAGTAGCTGCCATCGTGTG-3’ | 5’-TACTCCCCAAAGTCCACTTCG-3’ |
| *Lgr5* | NM_010195 | 5’-CACCCCAATGCGTTTTCTAC-3’ | 5’-GATGGTATCAGGCTCTGTAAGG-3’ |
| *Psors1c2* | NM_020576 | 5’-TCTCCCGGATCTACAGACACC-3’ | 5’-GGAGGTTCATCAAACAAAGGAGG-3’ |
| *Igf1r* | NM_010513 | 5’-GTGGGGGCTCGTGTTTCTC-3’ | 5’-GATCACCGTGCAGTTTTCCA-3’ |
| *Ltbp1* | NM_019919 | 5’-TGCCAGAACGGAGGGATGT-3’ | 5’-GGTTGACGTATGTTTTGCTGC-3’ |
| *Rhoc* | NM_007484 | 5’-ATGGCTGCGATCCGAAAGAAG-3’ | 5’-GCACGTAGACCTCTGGAAACT-3’ |
| *Ctgf* | NM_010217 | 5’-GGGCCTCTTCTGCGATTTC-3’ | 5’-ATCCAGGCAAGTGCATTGGTA-3’ |
| *Tyr* | NM_011661 | 5’-CTCTGGGCTTAGCAGTAGGC-3’ | 5’-GCAAGCTGTGGTAGTCGTCT-3’ |
| *Crnn* | NM_001081200 | 5’-AGAAACTTGTCCCATCCTGC-3’ | 5’-CTCTTCAACTCCTGCCTCG-3’ |
| *Krt31* | NM_010659 | 5’-GGTGCAGATAGATAATGCCAAGC-3’ | 5’-AGCTCATCCAAGATCCTTCGC-3’ |
| *Krap3-3* | NM_025524 | 5’-GCTCCCTTGCTTGGCACCAGT-3’ | 5’-TCCGAACTGCACACGGTCGGTA-3’ |

**Sequences of Primers Used for qPCR Experiments**
